# Sevoflurane concentration for cannulation in developmental disabilities

**DOI:** 10.1186/s12871-022-01695-5

**Published:** 2022-05-16

**Authors:** Naou Kunihiro, Masanori Tsukamoto, Shiori Taura, Takashi Hitosugi, Yoichiro Miki, Takeshi Yokoyama

**Affiliations:** 1 Kyushu University Graduate School of Dental Science, 3-1-1, Maidashi, Higashi-ku, Fukuoka, 812-8582 Japan; 2 Department of Dental Anesthesiology, Kyushu University Hospital, Fukuoka, Japan; 3 Department of Dental Anesthesiology, Graduate School of Dental Science, Kyushu University, Fukuoka, Japan; 4 Department of Dental Anesthesiology, Faculty of Dental Science, Kyushu University, Fukuoka, Japan; 5 Faculty of Arts and Science, Kyushu University, Fukuoka, Japan

**Keywords:** Sevoflurane concentration, Intravenous cannulation, Developmental disabilities

## Abstract

**Objective:**

The goal of this study was to compare the end-tidal sevoflurane concentration and time for intravenous cannulation at induction of anesthesia using sevoflurane with or without nitrous oxide in healthy children and in those with developmental disabilities.

**Methods:**

Normal and developmentally disabled children were anesthetized by inhalation of sevoflurane with nitrous oxide or with nitrous oxide-free oxygen, and intravenous cannulae were introduced. Nitrous oxide was stopped after loss of consciousness. The following parameters were recorded for each patient: age, gender, height, weight, BMI, duration of intravenous cannulation, end-tidal concentration of sevoflurane at the completion of intravenous cannulation, and use of nitrous oxide.

For each parameter except gender, *p*-value were calculated by one-way analysis of variance (ANOVA). For gender, *p*-value were calculated using the Fisher method. Two-way ANOVA was performed to evaluate the effect of patient health status and nitrous oxide use on the end-tidal concentrations of sevoflurane and the time required for intravenous cannulation.

**Results:**

The end-tidal sevoflurane concentrations at the completion of the intravenous cannulation had received a significant main effect of the factor "the use of nitrous oxide" (*F*(1,166) = 25.8, *p* < 0.001, η^2^ = 0.13) and a small effect of the factor "the patient health status" (*F*(1,166) = 0.259, *p* = 0.611, η^2^ = 0.001). However, the time required for intravenous cannulation was not significantly affected by either of the two factors, "the use of nitrous oxide" (*F*(1,166) = 0.454, *p* = 0.501, η^2^ = 0.003) and "the patient health status" (*F*(1,166) = 0.308, *p* = 0.579, η^2^ = 0.002).

**Conclusions:**

Between the healthy children and the children with developmental disabilities, no significant differences in the time required for the intravenous cannulation from the beginning of anesthetic induction. However, the end-tidal sevoflurane concentrations at the completion of the intravenous cannulation was significantly different. Sevoflurane in alveoli might be diluted by nitrous oxide.

**Supplementary Information:**

The online version contains supplementary material available at 10.1186/s12871-022-01695-5.

## Introduction

The induction of anesthesia using sevoflurane is a widely used technique for pediatric patients as it eliminates issues such as fear of needles, body movement and pain associated with intravenous cannulation [1–3]. Nitrous oxide is used with sevoflurane and oxygen in order to achieve faster loss of consciousness and reduced excitatory movements [2, 3]. Anesthesiologists have relied on clinical parameters such as the loss of eyelash reflex, heart rate and centralization of pupils to obtain the intravenous cannulation [1, 4]. Too early attempts to place an intravenous route immediately after the loss of consciousness may result in patient movement or respiratory complications such as laryngospasm and desaturation [2, 5, 6].

The developmental disabilities refer to a class of functional disorders in which affected persons require assistance, either mental or psychological [7, 8]. They commonly have cognitive impairments due to developmental delays or disorders such as cerebral palsy, epilepsy and autism. It is probable that general anesthesia may be affected by congenital conditions, such as abnormal neuronal activity in the brain. Therefore, the MAC of volatile anesthetics might be lower in children with developmental disabilities [9–12].

Previous reports have described the optimal timing for intravenous cannulation following sevoflurane inhalation for induction of anesthesia in pediatric patients using the up-and-down method or the loss of eyelid reflex [1–4]. There is to date, however, no literature comparing end-tidal sevoflurane concentration for intravenous cannulation between healthy children and those with developmental disabilities.

We hypothesized that the sevoflurane concentration for intravenous cannulation might differ between healthy children and those with developmental disabilities. The goal of this study was to compare the end-tidal sevoflurane concentration and time for intravenous cannulation at induction of anesthesia using sevoflurane with or without nitrous oxide in healthy children and in those with developmental disabilities.

## Methods

The Ethic Review Board of Kyushu University Hospital approved this retrospective study (Approval No. 30–352). The target patients were healthy children and children with developmental disabilities, aged 4 to 16 years, who underwent dental care and/or oral maxillofacial surgery in Kyushu University Hospital under general anesthesia from April 2012 to October 2018. Types of developmental disorders included attention-deficit hyperactivity disorder (ADHD), autism, developmental delays, mental retardation, cerebral palsy, seizure, other developmental delays. All anesthesia records of pediatric patients during the period were checked. This study excluded patients with a history of respiratory infection within the past two weeks as well as patients with a history of chronic respiratory disorder. The patients were transferred to the operating room without receiving any premedication. Anesthesia was then induced by inhalation of 8% of sevoflurane in 4 L/min of nitrous oxide and 2 L/min of oxygen or in 6 L/min of oxygen after the start of non-invasive monitoring of oxygen saturation by pulse oximetry (SpO_2_), electrocardiogram (ECG), and non-invasive blood pressure (BP) and heart rate (HR) checks. The total fresh gas flow was 6 L/min. After the loss of consciousness, inhalation of nitrous oxide was terminated and sevoflurane concentration was adjusted according to each patient’s hemodynamic conditions. In the case of airway obstruction, jaw-lift was applied to relieve the obstruction, and ventilation was gently assisted as necessary. Those settings were maintained until the intravenous cannula was introduced. A tourniquet was used to make the vessel enlargement, and the anesthesiologist tried the cannulation into the dorsum vein with a 22 or 24 gauge cannula. If the patient exhibited a reaction to the sting to the skin such as body movement, the concentration of sevoflurane was increased. The attempts for intravenous cannulation were continued until successful cannulation (defined as intravenous fluid dripping into the intravenous tubing) was achieved.

The following parameters were recorded for each patient: age, gender, height, weight, BMI, time for intravenous cannulation, end-tidal sevoflurane concentration at the completion of the intravenous cannulation, and the use of nitrous oxide.

For each parameter other than gender, a *p*-value was calculated by one-way analysis of variance (ANOVA) to test the null hypothesis that there is no difference in the mean of that parameter among the four groups. For gender, a *p*-value was calculated using the Fisher method to test the null hypothesis that there is no difference in the gender ratio. A two-way ANOVA was performed to evaluate the effects of the health condition of patients and the use of nitrous oxide on the end-tidal concentration of sevoflurane and the times required for intravenous cannulation.

All values were expressed as mean ± standard deviations (SD) or numbers (n). *p*-values lower than 0.01 were considered statistically significant (two-way ANOVA).

## Results

One hundred and seventy patients were suitable for the present study. Patients were divided into two groups, a group of healthy children and a group of children with developmental disabilities, and further divided by whether nitrous oxide was used or not in the anesthesia induction. No patient had respiratory complications such as laryngospasm and desaturation. Demographic data are shown in Table 1. Table 2 shows the end-tidal sevoflurane concentration (mean ± SD) at intravenous cannulation in each group, and Table 3 shows the time (mean ± SD) required for completion of the intravenous cannulation from the induction of anesthesia in each group. With regard to the end-tidal sevoflurane concentrations at the completion of the intravenous cannulation, ANOVA revealed a significant main effect of the factor "the use of nitrous oxide" (*F*(1,166) = 25.8, *p* < 0.001, η^2^ = 0.13) and a small effect of the factor "the patient health status" (*F*(1,166) = 0.259, *p* = 0.611, η^2^ = 0.001).

**Table 1:** Demographic data

	Healthy children	Developmental disabilities	Healthy children	Developmental disabilities	*p* value
	Non-Nitrous oxide	Non-Nitrous oxide	Nitrous oxide	Nitrous oxide	
	(*n* = 55)	(*n* = 55)	(*n* = 30)	(*n* = 30)	
Age (yrs)	6.9 ± 2.2	7.3 ± 2.5	7.0 ± 2.9	8.2 ± 2.8	0.189
Gender (M/F)	30/25	42/13	19/11	21/9	0.105
Height (cm)	116.8 ± 15.1	117.3 ± 13.3	117.0 ± 16.8	119.5 ± 17.2	0.884
Weight (kg)	22.2 ± 8.1	23.1 ± 7.3	23.5 ± 9.5	24.3 ± 15.9	0.810
BMI (kg/m^2^)	15.8 ± 1.8	16.3 ± 2.4	16.6 ± 2.2	15.9 ± 4.1	0.493

Data were expressed as the number of patients or mean ± standard deviation (SD)

**Table 2:** End-tidal sevoflurane concentration

	End-tidal sevoflurane concentration (%)	
	Nitrous oxide	Non-Nitrous oxide
Healthy children	4.1 ± 0.8	5.1 ± 1.1
Children with developmental disabilities	4.2 ± 0.9	4.9 ± 1.3

**Table 3:** Time required for intravenous cannulation

	Time required for intravenous cannulation (sec)	
	Nitrous oxide	Non-Nitrous oxide
Healthy children	346 ± 210	349 ± 153
Children with developmental disabilities	381 ± 266	339 ± 189

ANOVA also revealed that the time required for intravenous cannulation was not significantly affected by either of the two factors, "the use of nitrous oxide" (*F*(1,166) = 0.454, *p* = 0.501, η^2^ = 0.003) and "the patient health status" (*F*(1,166) = 0.308, *p* = 0.579, η^2^ = 0.002).

## Discussion

In this study, we assessed the effect of the two factors, the use of nitrous oxide and the patient health status, on the end-tidal sevoflurane concentrations at the completion of the intravenous cannulation and on the time required for intravenous cannulation. We found that the use of nitrous oxide had a significant effect on the end-tidal sevoflurane concentration but no effect on the time required for cannulation. It was also revealed that the patient health status had no significant effect on either of the two variables.

The optimal timing for intravenous cannulation after the loss of eyelash reflex is affected by various factors, such as the volume of the breathing system fresh gas flow rate, sevoflurane concentration in alveoli and ventilation / perfusion mismatch [1].

It has been reported that epileptiform activity during general anesthesia in children with developmental disabilities affect BIS values [10–12]. The ictal and interictal EEG activity such as δ waves might be different from those of healthy patients [9, 12]. BIS values were used only for the depth of anesthesia during surgery, not for induction of anesthesia. In addition, inhalational anesthetics might influence GABA receptors in the CNS. It has been shown that the MAC of inhalational anesthetics can be up to 20% lower in cerebral palsy patients [11]. However, there were no physiological signs or monitoring methods to determine the optimal timing of intravenous cannulation after loss of the eyelash reflex.

Anesthesiologists are usually anxious to obtain an intravenous route as soon as possible at induction of anesthesia in order to administer agents for faster intubation. Attempt of intravenous cannulation just after the loss of the eyelash reflex may lead to premature attempt for the patients, which may cause body movement, laryngospasm, attempt failure, and coughing [2].

Recently, ketamine was categorized into narcotic drugs legally, and was becoming not popular for pediatric patients in Japan. Ketamine and local anesthetic creams were not used in this study. On the other hand, sevoflurane is widely used inhalational anesthetic in pediatric anesthesia for mask induction. And nitrous oxide, used in this study, is a well-established acting analgesic and sedative agent [1, 2, 13]. We expected that the combination of sevoflurane and nitrous oxide was beneficial to make the optimum timing for intravenous cannulation shorter without causing complications [13, 14]. However, when stopping the admin of nitrou oxide, nitrous oxide enters the alveoli rapidly from the blood. The dilution of the alveolar air-gas mixture leads to a decrease in the concentration of alveolar sevoflurane. Subsequently the end-tidal sevoflurane concentration might be decreased. This phenomenon might counteract the benefit of nitrous oxide.

Compared to patients in previous studies, none of the patients in our study had hemodynamic and/or respiratory complications such as laryngospasm and desaturation. This could be explained by higher concentration of sevoflurane (5–8%) for anesthetic induction, which could ensure safe depth [1–4]. However, our retrospective results showed that the time for intravenous cannulation did not shorten in spite of nitrous oxide inhalation. We should not stop nitrous oxide until the venous route was obtained.

Our study has some limitations. Attempts of intravenous cannulation depend each anesthesiologist and their experiences. Due to the condition of blood vessels, cannulation could be difficult and it might take longer time. Also, the number of cannulation attempts could have been difficult to record. As a next step, further prospective studies are required with larger sample sizes, limited number of anesthesiologists and continuing nitrous oxide inhalation until securing venous cannulation.

## Conclusions

Between the healthy children and the children with developmental disabilities, there was no significant differences in the time for the intravenous cannulation from the beginning of anesthetic induction. When nitrous oxide was used for induction of anesthesia, on the other hand, the end-tidal sevoflurane concentrations at the completion of the intravenous cannulation decreased significantly in both children groups. In the case of termination of nitrous oxide after the loss of consciousness, alveolar concentration of sevoflurane might be diluted by nitrous oxide returned from the blood. Therefore, the time for the completion of the intravenous cannulation could not be shortened in both children groups. We should probably continue nitrous oxide until the completion of the intravenous cannulation for rapid intravenous cannulation.

## Supplementary Information

**Additional file 1.**

## Data Availability

All data generated or analysed during this study are included in this published article and its supplementary information files.
